# A Comparative Study on Flat and U-Shaped Copper Strips Produced by Continuous Extrusion

**DOI:** 10.3390/ma15134405

**Published:** 2022-06-22

**Authors:** Mo Zhou, Xinbing Yun, Hongwang Fu, Ying Zhang, Yuanwen Liu

**Affiliations:** 1 Engineering Research Center of Continuous Extrusion, Ministry of Education, Dalian Jiaotong University, Dalian 116028, China; zhoumo@djtu.edu.cn (M.Z.); hongwangfu@djtu.edu.cn (H.F.); yingzhang@djtu.edu.cn (Y.Z.); lyw@djtu.edu.cn (Y.L.); 2 College of Mechanical Engineering, Dalian University of Science and Technology, Dalian 116052, China

**Keywords:** continuous extrusion, expansion forming, processing parameters, copper strips, microstructure

## Abstract

The differences between flat and U-Shaped pure copper strips during a continuous extrusion process were investigated and analyzed through finite element simulation and experimentation. The simulation results showed that nearly all of the temperature-, velocity-, and loading-force-related parameters of the U-Shaped product at the die exit were smaller than those of the flat product, which indicated that extruding U-Shaped copper strips by continuous extrusion was superior to the flat strips. This conclusion was further verified experimentally by measuring the temperature and torque force. Then, a comparative analysis of the microstructure of the two cases was carried out. The average grain size of the U-Shaped strips was 65.6 µm, which was smaller than that of the flat strips, which was 96.7 µm. In addition, the microstructure of the U-Shaped strips was more uniform and had a higher recrystallization ratio, which can be attributed to the even and severe plastic deformation. This study thus solves the size limitation issue that existed in continuous extrusion.

## 1. Introduction

Continuous extrusion forming (CONFORM) is widely known to form strips or rods with low energy cost and good performance of the product [1]. In continuous extrusion, the metal in the groove suffers initiative friction from three sides, which drives the metal into the chamber and simultaneously generates friction heat to soften the material for better forming. This process needs no extra heating, thus saving energy. In addition, products with unlimited length can be manufactured, and the material utilization rate can reach more than 95% [2,3,4]. Therefore, continuous extrusion has been extensively applied for nonferrous alloys such as AM60 Mg [5], Al−Mg−Si [6], Cu−Te [7], Al−Fe−Cu [8], and Al−13Si−7.5Cu−Mg [9]. However, these investigations were mostly focused on the microstructure evolution and mechanical properties of small cross-sectional products, i.e., wires. Research on how to produce strips with a large width-thickness ratio has not been reported.

Copper strips with a large width–thickness ratio can be applied in many industrial fields. Traditional continuous casting can produce the products but suffers casting defects and non-uniform microstructure [10,11,12]. Traditional extrusion can produce high-quality strips, but the energy consumption is high, and the dimension of the product is limited [13,14]. Our research showed that flat copper strips with a cross-section of 300 mm × 14 mm can be produced by a TLJ 630 continuous extrusion machine (Dalian Konform Technical Company Ltd., Dalian, China). However, the flat strip exhibits a non-uniform microstructure due to an unevenly distributed temperature, as it is well known that high temperatures can lead to grain growth, resulting in coarse grains. Thus, controlling temperature distribution is a key issue in achieving a uniform microstructure for copper strips in continuous extrusion. In addition, extruding such a large cross-sectional flat strip needs a high loading force, which impairs the service life of the assembled tool and die structures. Therefore, the load force has to be reduced. Lastly, the industry needs a higher width–thickness ratio of the copper strip for manufacturing. In this study, we proposed a novel U-Shaped continuous extrusion process, and a comparative analysis of the U-Shaped (420 mm × 14 mm) and flat (300 mm × 14 mm) processes was performed, intending to solve the three aforementioned issues of continuous extrusion.

## 2. Experimental Details

**Figure 1:**
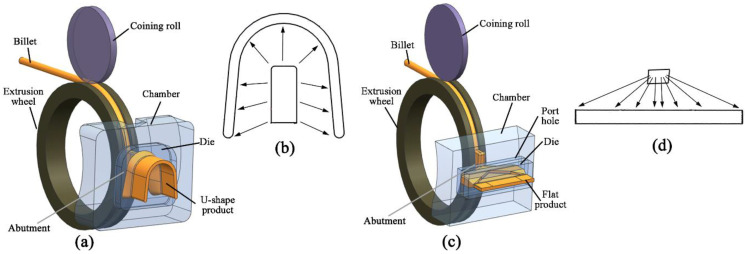
Schematic diagram of continuous extrusion: (**a**,**b**) U-Shaped strips; (**c**,**d**) Flat strips.

The continuous extrusion process with the newly designed expansion chamber and die for U-Shaped products is schematically shown in Figure 1a. For comparison, the continuous extrusion process of flat products is shown in Figure 1c. As depicted in Figure 1b, the distances from the outlet of the chamber to the exit of the die are approximately the same for U-Shaped products, whereas those of flat products are different, as shown in Figure 1d.

**Figure 2:**
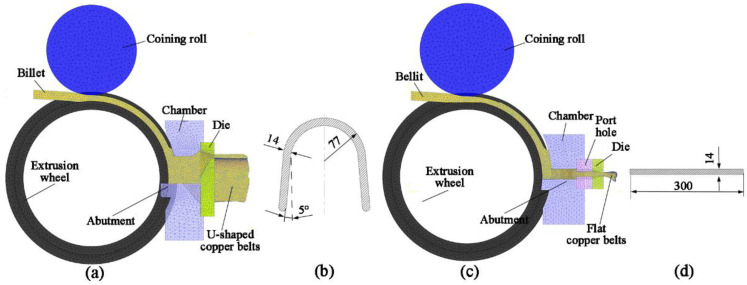
Finite element model and cross-sectional dimensions of copper strips: (**a**,**b**) U-Shaped strips; (**c**,**d**) Flat strips.

The continuous extrusion process of the U-Shaped and flat copper strips was simulated by DEFORM-3D 11.0, Columbus, OH, USA. The simulation mold of the U-Shaped strip is shown in Figure 2a, and the process for the flat strip is shown in Figure 2b. A tetrahedral element was employed for the copper billet in the simulation, and the number of elements is 49,270. As the mold for continuous extrusion is symmetrical, only half of the geometry was built in order to save time. The diameters of casting pure copper rods are both Φ30 mm, U-Shaped product size is 420 mm × 14 mm (Figure 2b), and flat size is 300 mm × 14 mm (Figure 2d). Table 1 shows the material properties and simulation parameters [15,16,17]. The distributions of temperature and velocity of metal in the continuous extrusion process were key parameters for the uniformity of the U-Shaped and flat strips. At the same time, the mean-square deviation of velocity (SDV), effective stress, extrusion wheel torque, and load of abutment were monitored and comparatively analyzed. For verification, experiments were carried out. A cast pure copper rod with a diameter of Φ30 mm was fed into the groove of the extrusion wheel, and the U-Shaped and flat copper strips were extruded by TLJ630. During these processes, the temperature and torque force of the extrusion wheel were recorded for comparison. Finally, the microstructure of the products was comparatively analyzed.

**Figure 3:**
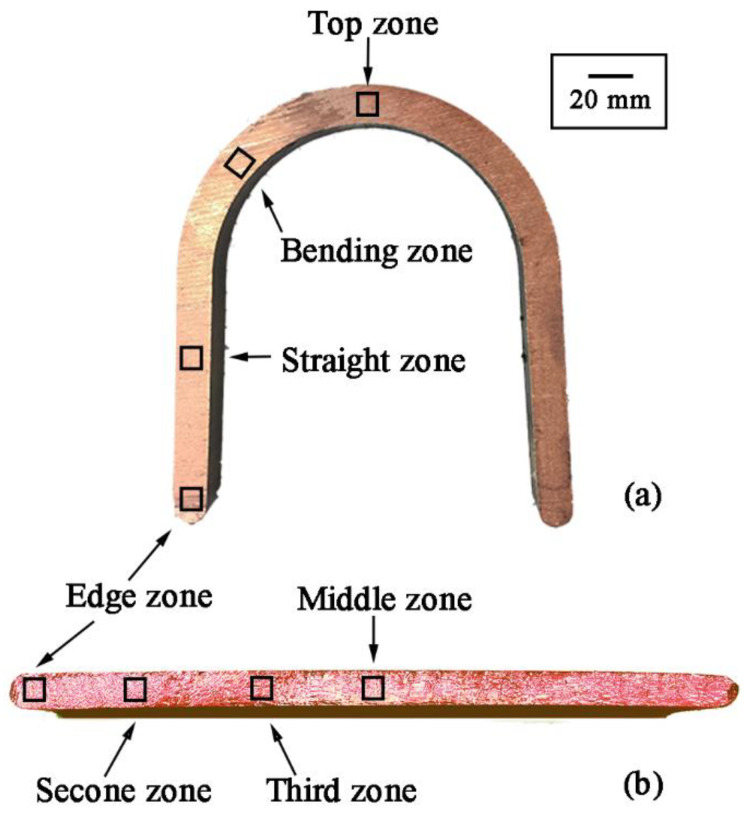
Areas marked on the cross-section of the product: (**a**) U-Shaped strips, (**b**) Flat strips.

For the U-Shaped copper strip, four zones, namely the edge zone, straight zone, bending zone, and top zone, were defined, as shown in Figure 3a. Correspondingly, those of the flat copper strip are marked by the edge zone, second zone, third zone, and middle zone, respectively (Figure 3b). Samples were cut from these zones for microstructure observation. For SEM measurement, the samples were thinned by ion milling, after which they were scanned by SUPRA 55 SAPPHIRE scanning electron microscopy (ZEISS, Jena, Germany) equipped with the electron backscatter diffraction (EBSD).

## 3. FEM Simulation Results and Discussion

### 3.1. Comparison of the Temperature

**Figure 4:**
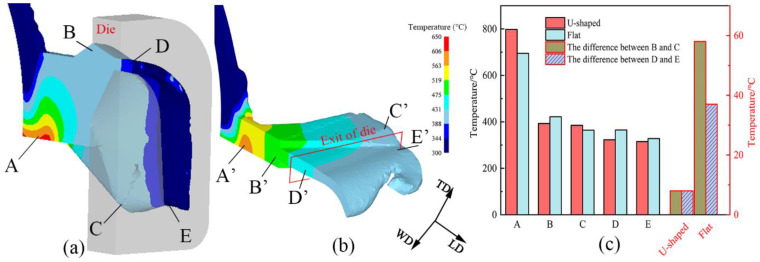
Distribution of simulated temperature of (**a**) U-Shaped and (**b**) Flat strips; (**c**) Temperature value and temperature difference.

The comparative study of U-Shaped copper strips and flat strips is discussed through FEM when the copper billet enters the stable deformation stage. Figure 4a,b show the marked areas chosen from the billet of U-Shaped and flat strips, respectively. The points A/A′, B/B′, C/C′, D/D′, and E/E′ are at the abutment, the top/middle of the outlet of the chamber, the bottom/edge of the outlet of the chamber, the top/middle of the die exit, and the bottom/edge of the die exit, respectively. The temperature variations of billets from chamber inlet to die exit exhibit a similar gradient trend. The temperature reaches a maximum at point A because of strong plastic deformation [18]. The maximum values are 798 °C for the U-Shaped strips and 695 °C for the flat strips. Figure 4c depicts the temperatures at points B, C, D, and E, which are 393, 385, 323, and 315 °C, respectively, and at points B’, C’, D’, and E’ are 422, 364, 365, and 328 °C, respectively. The temperature difference between B and C and D and E of the U-Shaped strips are both 8 °C, and those of flat strips are 58 and 37 °C, respectively. Although the temperatures at the abutment were slightly higher for the U-Shaped strips, the result favors the metal forming. As the extrusion proceeds, the temperature decreases to a comparable level to that of the flat strips. The temperature differences of U-Shaped strips are much lower at the chamber outlet and the die exit than those of the flat strips, however, indicating that the temperature of the U-Shaped strips is more homogenous.

### 3.2. Comparison of the Velocity

**Figure 5:**
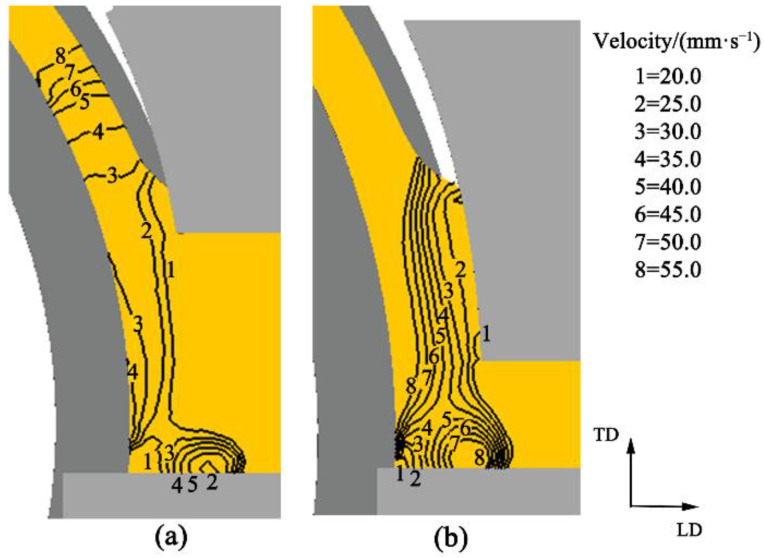
Distribution of velocity in the upsetting zone at the abutment: (**a**) U-Shaped strips; (**b**) Flat strips.

Former investigations indicated that flat strips with a small width–thickness ratio extruded by continuous extrusion normally exhibit a fast metal flow in the middle area but a slow metal flow at the edge area [19]. Therefore, the flow uniformity of the product during the continuous extrusion process is taken as the criterion to judge the extended forming. Figure 5 shows the metal flow from the wheel groove to the abutment (LD means the flow direction, and TD means the normal direction to flow direction). The flow direction changes when the metal hits the abutment and the metal at the groove next to it. Meanwhile, upsetting occurs at the back metal due to the resistance of the leading metal. Hence, the velocity is reduced in the vertical direction and accelerated at the right-angle bending area. However, there are differences between the U-Shaped and flat strips. The velocity contours of U-Shaped strips are nearly horizontal in the wheel groove and become upright when the metal enters the chamber outlet through the right-angle bending, as shown in Figure 5a. The velocity contours of flat strips are invariably upright, as depicted in Figure 5b. The difference between them is probably attributed to the height variation of the chamber outlet. When the billet leaves the right-angle bending zone, turbulent characteristics at the abutment are found for the two cases, i.e., a certain increase in velocity occurs. The increase in the U-Shaped strips is 15 mm/s, and that of the flat strips is 25 mm/s.

**Figure 6:**
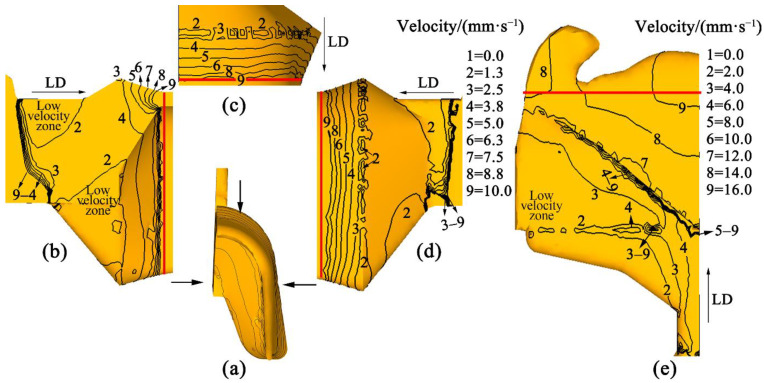
Distribution of velocity in the chamber and die: (**a**) U-Shaped strips; (**b**) On the symmetry plane of U-Shaped strips; (**c**) Planform of U-Shaped strips; (**d**) Right elevation of U-Shaped strips; (**e**) Planform of flat strips.

**Figure 7:**
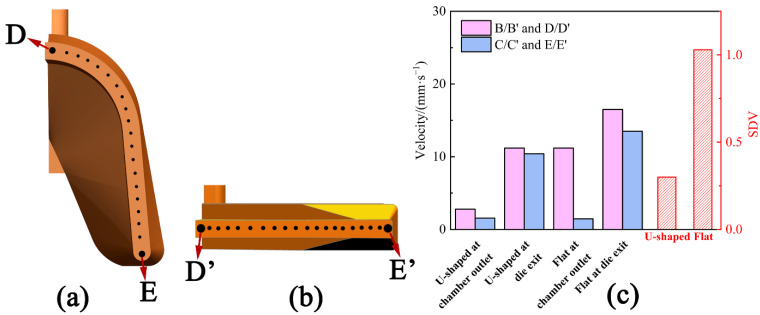
Characteristic points: (**a**) The exit of the U-Shaped die; (**b**) The exit of the flat die; (**c**) The velocity value and SDV.

The stable velocity entering chamber expansion of U-Shaped strips was 12.7 mm/s, and of the flat strips was 20.1 mm/s. Figure 6a,e show the velocity distributions of the U-Shaped and flat metal in the chamber and die, respectively. Figure 6b–d depicts the distribution of velocity in the U-Shaped strips from three views. The velocity contours decrease gradually at the entrance of the cavity because of the expansion of the metal [20]. In order to extrude the product smoothly, the metal expands sideways as held back by the die structure. Two low-velocity zones are displayed on the symmetry plane of the U-Shaped strips (Figure 6b) and one low-velocity zone on the flat strips (Figure 6e). Figure 7c depicts the velocity values (positions marked in Figure 4) and SDV of metal in the U-Shaped and flat strips at the chamber outlet and die exit. The velocity of the U-Shaped strips on the top of the outlet of chamber B point is 2.79 mm/s, and on the bottom C point is 1.55 mm/s. The velocity of the flat strips in the middle B’ point is 11.2 mm/s, and at the edge of the outlet of the chamber C’ point is 1.45 mm/s. The metal flow is faster near the die exit (marked with a red line in Figure 6b–e). However, the contours of the U-Shaped strips are perpendicular to the direction of extrusion, and those of the flat strips are at an angle to the direction of extrusion. The velocity of the metal of the U-Shaped strips on the top of the exit of the die D point is 11.2 mm/s, and on the bottom E point is 10.4 mm/s. The velocity of the flat strips in the middle D’ point is 16.5 mm/s, and at the edge of exit of chamber E’ point is 13.5 mm/s. Figure 7a shows 20 points of velocity at the die exit of the U-Shaped strips, and Figure 7b shows them for the flat strips. The SDV value was calculated by these points. The SDV value of the U-Shaped strips is 0.30, and that of the flat strips is 1.03, demonstrating that the metal of the U-Shaped product flows uniformly in the deformation process at the die exit. As shown in Figure 1, the metal filled in the chamber of the U-Shaped product expands in three-dimensional space, and the length of the routes from the chamber to each part of the die exit is similar, meaning that the metal arrives at the die exit with similar force and velocity. However, the metal for the flat strips expands only in the horizontal direction when it fills the chamber. The extension in the thickness direction is small, and the metal will suffer more resistance at the edge of the mold. Therefore, the metal in the middle area flows faster and slower at the edge.

### 3.3. Comparison of the Force

**Figure 8:**
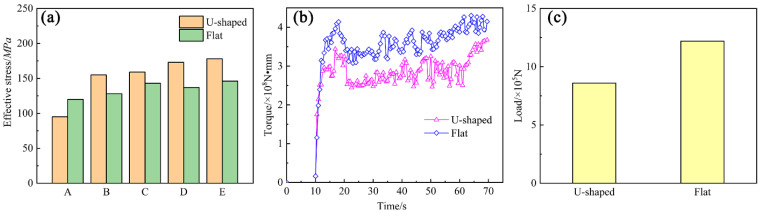
Simulated result: (**a**) Effective stress; (**b**) Torque of extrusion wheel; (**c**) Load of abutment.

Figure 8a represents the effective stress values of U-Shaped and flat metal at points A, B, C, D, and E marked in Figure 4a,b, which are 95, 155, 159, 173, and 178 MPa, respectively. Those of the flat strips are 120, 128, 143, 137, and 146 MPa, respectively. The effective stress differences of the chamber and die of the U-Shaped copper strips are 4 and 5 MPa, while those of the flat strips are 15 and 9 MPa, respectively, demonstrating that the metal of the U-Shaped suffered more uniform force during the continuous extrusion. Figure 8b depicts the torque of extrusion wheel variation with time, and the value after extrusion stability of U-Shaped strips is 4.0 × 10^8^, and that of the flat strips is 5.7 × 10^8^ N∙mm. Figure 8c shows the load of abutment in the vertical direction. The value of the U-Shaped strips is 8.6 × 10^5^, and that of the flat strips is 12.2 × 10^5^ N. From Figure 8b,c, it can be concluded that the extrusion force that the U-Shaped strips needed is lower than that needed by the flat strips.

## 4. Experimental Results and Analysis

### 4.1. Temperature and Load Measurement

**Figure 9:**
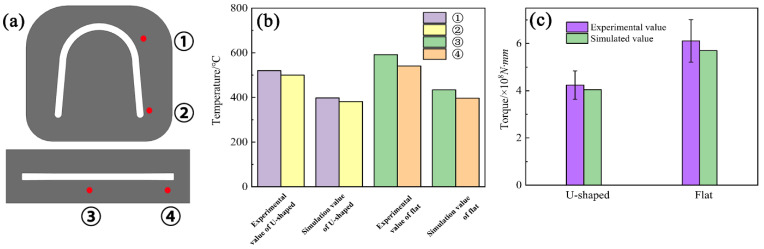
Experimental value and simulated value: (**a**) The temperature measuring points at the exit of the die; (**b**) Temperature; (**c**) Torque of the extrusion wheel.

Figure 9 depicts the measured positions, measured temperature, and torque force of the U-Shaped and flat strips during the continuous extrusion process. Two positions were chosen at the exit of the U-Shaped and flat dies, respectively, for temperature measuring, as revealed in Figure 9a. Figure 9b shows that the measured and simulated temperatures of the U-Shaped strips are 520, 500 °C, and 398, 382 °C, respectively, while those of the flat strips are 592, 541 °C, and 434, 397 °C, respectively. Figure 9c displays the measured and simulated torque force of the extrusion wheel of the U-Shaped and flat strips. The large temperature difference in the experiment is due to a measuring error. When the metal flowed at the die exit, the temperature was measured at the die instead of the metal. Moreover, the metal at the edge of the die would transfer more heat to the mold, but the mold will take away the same amount of heat from each area of the metal in an ideal simulation environment. The experimental and simulated torques of the U-Shaped strips are 4.2 × 10^5^ N∙m and 4.0 × 10^5^ N∙m, respectively, while those of the flat strips are 6.1 × 10^5^ N∙m and 5.1 × 10^5^ N∙m, respectively. The results show that both the measured and simulated temperatures and torque forces of the U-Shaped strips are smaller than those of the flat strips, indicating that the newly designed U-Shaped continuous extrusion is superior to the conventional flat process.

### 4.2. Microstructure

**Figure 10:**
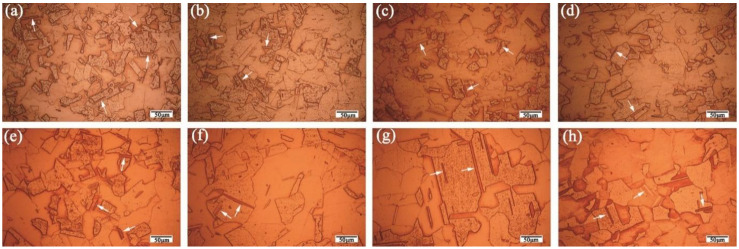
(**a**–**d**) Microstructures of four zones for U-Shaped strips. (**e**–**h**) Microstructures of four zones for flat copper strips: (**a**) Edge zone of U-Shaped strips; (**b**) Straight flange zone; (**c**) Bending zone; (**d**) Top zone; (**e**) Edge zone of flat strips; (**f**) Second zone; (**g**) Third zone; (**h**) Middle zone.

Figure 10 displays the microstructures of the U-Shaped and flat copper strips. Large numbers of annealing twins (marked with white arrows) and clean grain boundaries are found in each zone of the U-Shaped and flat strips. The average grain sizes of the edge zone (Figure 10a), straight flange zone (Figure 10b), bending zone (Figure 10c), and top zone (Figure 10d) of U-Shaped strips are 61.5, 65.2, 68.8, and 66.7 µm, respectively. Therefore, the average grain size of the U-Shaped copper strips is 65.6 µm. The distributions of grain sizes of the flat strips are also crystallographic homogeneous; nonetheless, the grain sizes are obviously larger than those of the U-Shaped ones. The average grain size of the edge zone (Figure 10e), second zone (Figure 10f), third zone (Figure 10g), and middle zone (Figure 10h) is about 70.6 µm, 98.6 µm, 115.1 µm, and 102.3 µm, respectively. The average grain size of flat strips is 96.7 µm. The coarse and unevenly distributed grains in the flat strips are due to the inhomogeneous temperature and plastic deformation during the continuous extrusion process.

**Figure 11:**
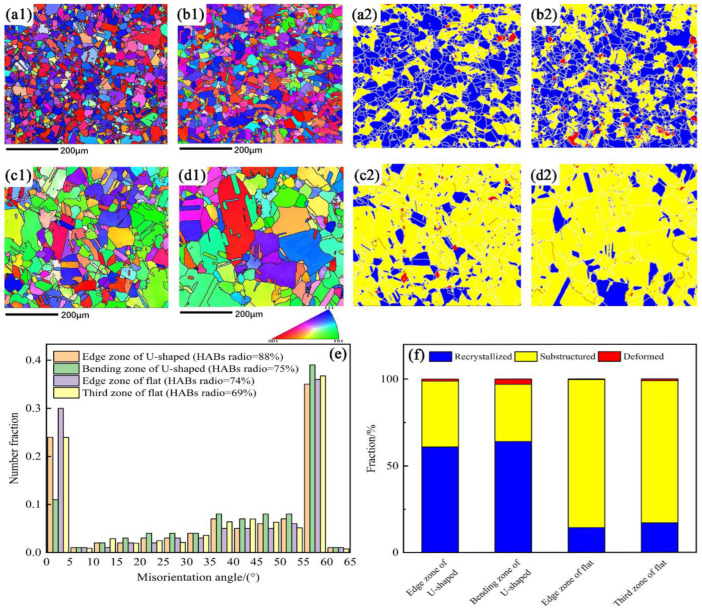
(**a1**–**d1**) EBSD patterns. (**a2**–**d2**) Maps of recrystallized, substructure, and deformed grains: (**a1**) Edge zone of U-Shaped strips; (**b1**) Bending zone; (**c1**) Edge zone of flat strips; (**d1**) Third zone; (**e**) Misorientation angle distribution; (**f**) Recrystallized, substructure, and deformed grain ratio.

To better display the microstructure information, EBSD analyses were carried out for two selected zones of the strips. Figure 11 represents the EBSD pattern, misorientation angle distribution, and maps of recrystallized, substructure, and deformed grains of the U-Shaped and flat strips. Here, grain boundary misorientation angles higher than 15° are defined as high-angle boundaries (HABs), and those lower than 15° and higher than 2° are defined as low-angle boundaries (LABs) [21]. As depicted in Figure 11(a1–d1), the grain size of the U-Shaped strips at the two zones is finer than that of the flat ones. Figure 11e displays the misorientation angle distribution. The HABs ratios for the two zones in the U-Shaped strips are 88% and 75%, respectively, which are higher than those of the flat strips ( 74% and 69%), respectively.

Figure 11(a2–d2) show the recrystallized, substructured, and deformed grains corresponding to Figure 11(a1–d1), respectively. The volume fraction of recrystallized grain in the edge and bending zones of the U-Shaped strips is 60.9% and 64.0%, respectively, while that of the edge and third zones of the flat strips is 14.3% and 17.0%, respectively. The volume fraction of substructured and deformed grain ratio in the U-Shaped strips is correspondingly smaller than that of the flat strips. As discussed previously, high temperature in continuous extrusion can lead to dynamic recrystallization. Although the temperature in the U-Shaped continuous extrusion process is lower, the volume fraction of recrystallized grains is greater than that of the flat process. The reason for that can be attributed to severe plastic deformation occurring during the U-shape forming process, which provides sufficient dislocations for recrystallization [22,23,24]. As the temperature of the flat strips is higher than that of the U-Shaped strips, the grains of the flat strips grow significantly. That is why the grain size of the flat strips is bigger than that of the U-Shaped strips. In addition, the temperature difference of flat strips is also more inhomogeneous, which leads to a larger difference in grain size between different areas.

## 5. Conclusions

The simulation results showed that nearly all of the temperature, temperature difference, velocity, and loading force-related parameters of the U-Shaped product at the die exit are lower than those of the flat product, which indicated that extruding U-Shaped copper strips by the continuous extrusion process is superior to that of flat strips.

The experiments performed proved that the temperature and torque of the extrusion wheel of the U-Shaped strips were lower than those of the extruded flat strips. Comparative analysis of microstructure indicated that the average grain size of the U-Shaped strips is 65.6 µm, which is smaller than that of the flat strips (96.7 µm). In addition, the U-Shaped copper strips have a more uniform microstructure and recrystallization ratio.The present work showed that the U-Shaped forming can solve the size limitation issue in continuous extrusion and also possesses the advantages of finer grain size, more uniform microstructure, and less loading force than those of flat forming.

## Figures and Tables

**Table 1:** Material properties and simulation parameters.

Billet Material	Mold Material	Extrusion Wheel Diameter/mm	Rotational Speed of Extrusion Wheel/(r·min^−1^)	Billet Diameter/mm	Temperature/°C			Friction Coefficient		
					Billet	Extrusion Wheel	Die	Billet and Extrusion Wheel	Billet and Chamber	Billet and Die
Pure copper	AISI-H-13	630	2.5	30	20	450	450	0.95	0.3	0.3

## Data Availability

Not applicable.
